# Contribution of Alcohol to Hypertension Mortality in Russia

**DOI:** 10.1155/2014/483910

**Published:** 2014-02-23

**Authors:** Y. E. Razvodovsky

**Affiliations:** Grodno State Medical University, Gorky Street 80, 230008 Grodno, Belarus

## Abstract

*Background*. Hypertension (HTN) is reported to be the leading contributor to premature death globally. Considerable research evidence suggests that excessive alcohol intake (binge drinking) is an independent risk factor for HTN. It was repeatedly emphasized that binge drinking is a major contributor to a high cardiovascular mortality rate in Russia. *Objective*. The aim of this study was to examine the aggregate-level relation between alcohol consumption and HTN mortality rates in Russia. *Method*. Age-standardized sex-specific male and female HTN mortality data for the period 1980–2005 and data on overall alcohol consumption were analyzed by means of ARIMA (autoregressive integrated moving average) time-series analysis. The level of alcohol consumption per capita has been estimated using the indirect method based on alcohol psychoses incidence rate and employing ARIMA time-series analysis. *Results*. Alcohol consumption was significantly associated with both male and female HTN mortality rates: a 1-liter increase in overall alcohol consumption would result in a 6.3% increase in the male HTN mortality rate and in a 4.9% increase in female HTN mortality rate. The results of the analysis suggest that 57.5% of all male HTN deaths and 48.6% of all female HTN deaths in Russia could be attributed to alcohol. *Conclusions*. The outcomes of this study provide support for the hypothesis that alcohol is an important contributor to the high HTN mortality rate in the Russian Federation. The findings from the present study have important implications with to regards HTN mortality prevention, indicating that a restrictive alcohol policy can be considered as an effective measure of prevention in countries with a higher rate of alcohol consumption.

## 1. Introduction

Hypertension (HTN) is reported to be the leading cause of mortality to which 13% of global deaths are attributed [1, 2]. Raised blood pressure (BP) has been identified as a major risk factor for coronary heart disease and stroke [2]. Therefore, prevention and controlling HTN is one of the most cost-effective strategies for reducing the global burden of cardiovascular disease morbidity and deaths [1].

Considerable research evidence strongly supports the concept that alcohol is a major risk factor for development of hypertension [3–9]. In the WHO Global Burden of Disease Study, 16% of all cases of HTN were attributable to alcohol [10]. Many population studies found evidence for a positive and linear association between alcohol consumption and incidence of HTN [4, 7, 11–15]. The Kaiser-Permanente Health Screening Survey involving more than 80.000 persons aged 15 to 75 years showed an average rise of 1 millimeter mercury systolic pressure for each standard drink per day [13]. A meta-analysis of the prospective studies reported a 40% increase in the relative risk of developing HTN in those drinking more than 25 g/day and a greater than fourfold increase risk in those drinking more than 100 g/day [6]. In subjects drinking 30 g/day or more, 20% of the incident cases of HTN were attributed to alcohol consumption [6].

Russia is a country with a high prevalence of HTN [16–18]. According to the results of BP measurement, the prevalence of HTN among Russian men and women was 57% and 55% respectively [19]. The liner dose-dependent relationships between alcohol consumption and BP [5, 20], together with high overall alcohol consumption [21–23], translate into a high alcohol-attributable risk for HTN in Russia. In a recent study the odds rations of high BP for Russian men consuming >1.2 liters of alcohol per year were 2.35 (95% CI 1.51–3.67) compared to nondrinkers [24]. There is also evidence that in Russia alcohol is linked to such outcomes of HTN as stroke deaths [25].

The calculation of alcohol-attributable mortality currently is routinely applied to provide an indication of the public health effect of harmful alcohol consumption for developing strategy to prevent alcohol-related mortality [26]. The alcohol-attributable fraction (AAF) is generally defined as the proportion of the disease that would not occur if lifetime exposure to alcohol was hypothetically changed to the counterfactual level of zero [26].

Swedish researcher Norström has suggested that aggregate data may be used for estimation of alcohol-attributable mortality and developed a method for AAF estimation based on time-series analysis of mortality rate and the level of overall alcohol consumption using ARIMA time-series analysis [27]. It was shown that the effect of alcohol on alcohol-related mortality estimated from time-series data was consistent with the corresponding ecological estimates [27].

The aim of the present study was to estimate the premature HTN mortality attributable to alcohol in Russia using aggregate-level data of HTN mortality and estimates of the overall level of alcohol consumption from 1980 to 2005.

## 2. Methods

### 2.1. Data

The data on age-adjusted sex-specific HTN mortality rates per 1000.000 of the population are taken from the Russian State Statistical Committee (*Rosstat*). The Rosstat's cause of death classification has undergone several changes in recent decades. Until 1988 the cause of death classification was based on the Soviet nomenclature which had a limited number of causes of death in comparison with the International Classification of Diseases (ICD) system. From 1989 to 1998 Rosstat used a coding scheme that was based on ICD-9. From 1999 a new coding system based on ICD-10 was introduced. Rosstat issued a table of correspondence between its classification system and ICD-9 and ICD-10 and it has been claimed that the Russian system of coding was and is compatible with the ICD. For example, Soviet classification 400.09–404.99 “hypertensive disease” corresponds with ICD-9 code 401.0–405.11 and with ICD-10 code I10.9–I15.9.

Realizing the difficulties associated with measuring alcohol consumption at the population level in Russia [23] we employed an alternative measure of overall alcohol consumption relative to Nemtsov's estimates [21]. Estimation of alcohol consumption per capita was based on a set of indicators of alcohol-related harm which was adjusted for the effect of recorded alcohol consumption employing ARIMA (autoregressive integrated moving average) model [28]. More specifically, we calculated the level of unrecorded alcohol consumption as the difference between observed changes in the harm indicator and changes that would be expected on the basis of alcohol sales. The harm indicator series used was alcohol psychoses incidence rate because this indicator depends almost entirely on alcohol consumption [22]. We specified the number of persons, witches were admitted for the first time for the treatment as incidence of alcohol psychoses: (ICD-10: F 10). The data on alcohol psychoses incidence rate (per 100.000 of the population) are taken from the Russian State Statistical Committee (*Rosstat*). The alcohol series used was the volume of alcohol sales in liters of pure alcohol per capita. The data on alcohol sales per capita are taken from the Russian State Statistical Committee (*Rosstat*).

### 2.2. Statistical Analysis

To examine the relation between changes in the alcohol consumption and HTN mortality across the study period a time-series analysis was performed using the statistical package “Statistica.” The dependent variables were the annual HTN mortality and the independent variable was aggregate overall alcohol consumption. Bivariate correlations between the raw data from two time-series can often be spurious due to common sources in the trends and due to autocorrelation [29]. One way to reduce the risk of obtaining a spurious relation between two variables that have common trends is to remove these trends by means of a “differencing” procedure, as expressed in formula:

(1) $$\begin{array}{c} \nabla x_{t}=x_{t}-x_{t-1}. \end{array}$$

This means that the annual changes “∇” in variable “*X*” are analyzed rather than raw data. The process whereby systematic variation within a time-series is eliminated before the examination of potential causal relationships is referred to as “prewhitening.” This is subsequently followed by an inspection of the cross-correlation function in order to estimate the association between the two prewhitened time-series. It was Box and Jenkins [30] who first proposed this particular method for undertaking a time-series analysis and it is commonly referred to as ARIMA modeling. We used this model specification to estimate the relationship between the time-series HTN mortality and alcohol consumption rates in this paper. In line with previous aggregate studies [29, 31–33], we estimated semilogarithmic models with logged output. The following model was estimated:

(2) $$\begin{array}{c} \nabla \ln M_{t}=a+\beta \nabla A_{t}+\nabla N_{t}, \end{array}$$

where ∇ means that the series is differenced, *M* is HTN mortality rates, *a* indicates the possible trend in HTN mortality due to other factors than those included in the model, *A* is the alcohol consumption, *β* is the estimated regression parameter, and *N* is the noise term. The percentage increase in HTN mortality rates associated with a 1-liter increase in alcohol consumption is given by the expression: (exp⁡(*β*_1_) − 1)∗100. The temporal structure of the error term was estimated by using autoregressive (AR) or moving average (MA) parameters in the model. A diagnostic test for residual correlation is given by the Box-Ljung *Q*-test, which indicates whether the model has been adequately fitted.

In addition to the estimated effect parameter, the alcohol effect will also be expressed in terms of AAF, which can be calculated from the estimates obtained in ARIMA models according to the following formula: AAF = 1 − exp⁡(−*bX*), where *X* is alcohol consumption for the whole study period and *b* is the estimated effect parameter [27].

## 3. Results

**Figure 1:**
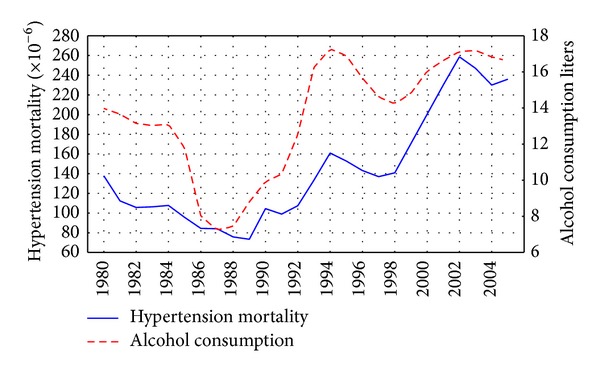
Trends in male HTN mortality rate and alcohol consumption per capita in Russia between 1980 and 2005.

**Figure 2:**
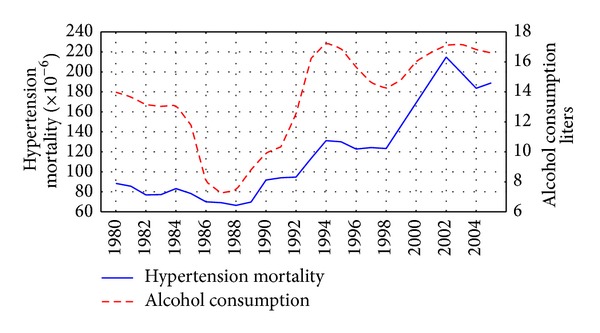
Trends in female HTN mortality rate and alcohol consumption per capita in Russia between 1980 and 2005.

The trends in the age-adjusted, sex-specific HTN mortality rates are displayed in Figures 1 and 2. For both sexes the time-series HTN mortality rates fluctuated greatly over the period, decreased markedly between 1984 and 1988, then started on an upward trend from 1988 to 1989, before increasing substantially during 1992 to 1994 (by 49.7% and 38.5% for men and women, resp.). From 1995 to 1998 there was a fall in the rates before they again jumped dramatically between 1998 and 2002 (by 83.7% and 74.2% for men and women, resp.), while a decrease in rates has been recorded in recent years.

The graphical evidence suggests that the trends in both alcohol consumption per capita and HTN mortality for males and females seem to follow each other across the time-series (Figures 1 and 2). As can be seen, there were sharp trends in the time-series data across the study period. These trends were removed by means of a first-order differencing procedure. After prewhitening the cross-correlations between alcohol consumption and the HTN mortality time-series were inspected. This indicated that there was a statistically significant cross-correlation between alcohol consumption and HTN mortality for males and females at lag 0 (Table 1). The specification of the bivariate ARIMA model and outcome of the analyses are presented in Table 2. According to the results, alcohol consumption is statistically significant and is associated with both male and female HTN mortality rates, implying that a 1-liter increase in per capita consumption is associated with an increase in male mortality of 6.3% and female mortality of 4.9%. Table 2 shows the relative proportion of alcohol-attributable deaths to all HTN deaths by gender. The results of the analysis suggest that 57.5% of all male deaths and 48.6% of female deaths from HTN in Russia could be attributed to alcohol.

## 4. Discussion

According to the results of time-series analysis there was a positive and statistically significant effect of per capita alcohol consumption on HTN mortality in Russia at lag zero. We argue that in this case the independent variable (per capita alcohol consumption) directly influenced the dependent variable (HTN mortality) and there is no evidence of a lagged relationship between the two time-series. These findings clearly indicate that population drinking and HTN mortality are positively related phenomena in Russia. The results of the present study are important because despite the growing literature on alcohol and mortality in Russia there has been no prior time-series analysis of alcohol and HTN mortality in the country. The positive and significant contemporaneous association between alcohol and HTN mortality in the present analysis replicates the recent findings from a time-series study highlighting the close temporal association between alcohol and ischemic heart disease mortality in Russia [32].

There is evidence that the HTN mortality trends in Russia are influenced by the three major factors: Gorbachev's antialcohol campaign 1985–1988; severe socioeconomic crisis imposed by rapid societal transformation in the early 1990s; and financial crisis and a worsening economic situation in 1998. A fairly close match between trends in alcohol consumption and HTN mortality during the Gorbachev's antialcohol campaigns may be used as evidence for the hypothesis suggesting that alcohol is responsible for a substantial number of HTN deaths in Russia. This empirical evidence also indicates that a restrictive alcohol policy can be considered as an effective measure in HTN mortality prevention.

It seems plausible that alcohol is a key variable in explaining the increase in the HTN mortality rate in the early 1990s. An increase of alcohol consumption in this period was to a great extent due to an increase of alcohol availability following the repeal of the state alcohol monopoly in January 1992 [21]. There are several potential factors behind the decrease in alcohol consumption and the HTN mortality rate between 1994 and 1998. They include better regulation of the alcohol market that may have resulted in a relative increase in prices for vodka compared to those for food products [22]. Another possible factor in the decrease in alcohol consumption was impoverishment and a decrease in the purchasing capacity of the population was due to unpaid or delayed salaries [21]. The subsequent rise in alcohol consumption and HTN mortality rates from 1998 may be associated with the financial crisis which resulted in hyperinflation, increasing poverty, and political and economic uncertainty [22].

Before concluding, several potential limitations of this study must be mentioned. In particular, there was the risk of omitted variable bias in this work. A number of modifiable risk factors for HTN have been identified including psychosocial distress, smoking, obesity, high salt intake, and sedentary lifestyle [2, 34]. Some experts have underlined the importance of the effect of the psychosocial distress of economic and political reforms as the main reason for the cardiovascular mortality crisis in Russia in the early 1990s [35]. In this period Russia faced a deep socioeconomic crisis accompanied by an increase in unemployment, hyperinflation, and a dramatic decline in the well-being of the majority of the population [22]. The turmoil associated with socioeconomic and political transition affected Russian people and led to the relatively high prevalence of depression, anxiety, and sleeping disorders that were strongly associated with low socioeconomic status, poor nutrition, and adverse health behavior such as binge drinking and smoking [36]. It might be the case that psychosocial distress was the main cause of an increased demand for alcohol at this time. This demand was met by factors that increased supply following the repeal of state alcohol monopoly in 1992. Empirical evidence also suggests that psychosocial distress imposed by the financial crisis has played a crucial role in the Russian HTN mortality crisis between 1998 and 2002 [22]. So, psychosocial distress may be an important underlying factor in HTN mortality fluctuation in Russia during the last decades.

It is, however, possible that both psychosocial distress and alcohol relations to HTN mortality at the aggregate level are spurious and there are other powerful risk factors. Cigarette smoking has been estimated to be a powerful modifiable risk factor of HTN mortality and also to be correlated with alcohol consumption [37]. The high prevalence of smoking among Russian men [38] probably explains a part of the high male HTN mortality rate compared with female mortality rate. However, there is little evidence of rapid changes in smoking patterns among Russian men and women that have translated into a substantial growth of HTN mortality in the 1990s [22]. Furthermore, use of tobacco products was relatively stable during the 1970s–1980s and has fallen substantially in Russia during the 1990s [39], suggesting that cardiovascular mortality crisis is not a result of a long-term response to smoking trends. Thus, playing the major causal role, smoking may represent a confounding factor.

Finally, the estimates of AAF for women, where heavy drinking is restricted to a relatively small proportion of the population gives rise to the suspicion of possible measurement error. It should be recognized that ignoring the confounding variables may imply that the alcohol effect is overestimated. Nevertheless, there are some indications that Russian women are drinking more now which is likely to be a factor in the narrowing of the male-female alcohol-related mortality rate ratio [23]. In his recent study, based on the results of RLMS, Perlman highlighted that frequent heavy drinking almost doubled among women between 1994 and 2004 [40].

An alternative explanation for these findings is that women are more sensitive to an increased risk of HTN, even at the relatively low levels of alcohol consumption. Indeed, in a systematic review of studies of moderate alcohol consumption in relation to blood pressure Burger et al. concluded that there were linear BP elevations at drinking levels of >20 g/day for women and >30 g/day for men [41]. Furthermore, in women the effects of alcohol use appear to be additive to that of the BP-rising effects of contraceptives [42].

In conclusion, this is the first time-series analysis of the overall level of alcohol consumption and HTN mortality rate in Russia, which has shown that population drinking is a strong predictor of HTN mortality rate at the aggregate level. The outcomes of this study provide indirect support for the hypothesis that the high rate of HTN mortality in Russia may be related to alcohol, as indicated by a close aggregate-level association between number of deaths from HTN and alcohol consumption per capita. The findings from the present study have important implications with regards to cardiovascular mortality prevention, indicating that a restrictive alcohol policy can be considered as an effective measure of prevention in countries with a higher rate of alcohol consumption per capita.

## Figures and Tables

**Table 1:** The results of cross-correlation analysis between prewhitened time-series. Effects of alcohol consumption per capita on HTN mortality rates.

Lag	Mortality males		Mortality females	
	*r*	SE	*r*	SE
−3	−0.191	0.213	−0.271	0.213
−2	0.052	0.209	0.061	0.209
−1	0.313	0.204	0.324	0.204
0	0.591	0.200	0.567	0.200
1	0.332	0.204	0.167	0.204
2	0.050	0.209	0.036	0.209
3	−0.071	0.213	0.048	0.213

**Table 2:** Estimated effects (bivariate ARIMA model) of overall alcohol consumption on IHD mortality rates.

Parameter	Model	Estim.	*P*	AAF
HTN mortality males	0.1.1*	0.063**	0.000	0.575
HTN mortality females	0.1.1	0.049	0.001	0.486

*The general form of nonseasonal ARIMA model is (*p*, *d*, *q*), where *p* is the order of the autoregressive parameter, *d* is the order of differencing, and *q* is the order of the moving average parameter. *Q*-test for residuals are satisfactory in all models.

**Estimates express the actual alcohol effect/100.
